# *In Silico* Analysis of the Small Molecule Content of Outer Membrane Vesicles Produced by *Bacteroides thetaiotaomicron* Indicates an Extensive Metabolic Link between Microbe and Host

**DOI:** 10.3389/fmicb.2017.02440

**Published:** 2017-12-08

**Authors:** William A. Bryant, Régis Stentz, Gwenaelle Le Gall, Michael J. E. Sternberg, Simon R. Carding, Thomas Wilhelm

**Affiliations:** ^1^Centre for Integrative Systems Biology and Bioinformatics, Department of Life Sciences, Imperial College London, London, United Kingdom; ^2^Gut Health and Food Safety Programme, Quadram Institute Bioscience, Norwich, United Kingdom; ^3^Metabolomics Unit, Quadram Institute Bioscience, Norwich, United Kingdom; ^4^Norwich Medical School, University of East Anglia, Norwich, United Kingdom; ^5^Theoretical Systems Biology Lab, Quadram Institute Bioscience, Norwich, United Kingdom

**Keywords:** *Bacteroides thetaiotaomicron* VPI-5482, outer membrane vesicle, metabolomics, genome-scale metabolic modeling, host–microbe interaction

## Abstract

The interactions between the gut microbiota and its host are of central importance to the health of the host. Outer membrane vesicles (OMVs) are produced ubiquitously by Gram-negative bacteria including the gut commensal *Bacteroides thetaiotaomicron*. These vesicles can interact with the host in various ways but until now their complement of small molecules has not been investigated in this context. Using an untargeted high-coverage metabolomic approach we have measured the small molecule content of these vesicles in contrasting *in vitro* conditions to establish what role these metabolites could perform when packed into these vesicles. *B. thetaiotaomicron* packs OMVs with a highly conserved core set of small molecules which are strikingly enriched with mouse-digestible metabolites and with metabolites previously shown to be associated with colonization of the murine GIT. By use of an expanded genome-scale metabolic model of *B. thetaiotaomicron* and a potential host (the mouse) we have established many possible metabolic pathways between the two organisms that were previously unknown, and have found several putative novel metabolic functions for mouse that are supported by gene annotations, but that do not currently appear in existing mouse metabolic networks. The lipidome of these OMVs bears no relation to the mouse lipidome, so the purpose of this particular composition of lipids remains unclear. We conclude from this analysis that through intimate symbiotic evolution OMVs produced by *B. thetaiotaomicron* are likely to have been adopted as a conduit for small molecules bound for the mammalian host *in vivo*.

## Introduction

The mammalian gastrointestinal tract (GIT) harbors a vast number and diversity of microbes which through extensive co-evolution have come to play a vital role in mammalian growth and health, particularly in both the development and maintenance of the immune system and in mammalian metabolism, previously reviewed extensively (Round and Mazmanian, 2009; Clemente et al., 2012). The intestinal microbiota degrades host-indigestible materials such as plant polysaccharides, maintains the structural integrity of the intestinal mucosal barrier, and protects against pathogen invasion. Fundamental to maintaining host–microbe relationships and a healthy GIT is effective cross-kingdom communication. As direct contact between luminal microbes and host cells in the GIT is highly restricted and normally prevented by a sterile mucus layer coating boundary epithelial cells (Johansson et al., 2008) effective communication between members of the microbiota and host cells relies on diffusible soluble mediators that can be delivered as free secreted products or contained within microvesicles such as outer membrane vesicles (OMVs) produced predominantly by Gram-negative bacteria (Stentz et al., 2014). A review of the functions and biogenesis of OMVs from Gram-negative bacteria was published recently (Schwechheimer and Kuehn, 2015).

One of the most abundant phyla present in the intestinal microbiota of both mouse and human is *Bacteroides* of which *B. thetaiotaomicron* VPI-5482 (Bt) is a prominent member and is used as a model in studies of host–bacterium interactions (Eckburg et al., 2005). Bt contributes to carbohydrate fermentation and the production of a pool of volatile fatty acids that are reabsorbed through the large intestine and utilized by the host as an energy source, providing a significant proportion of the host’s daily energy requirement (Hooper et al., 2002). Characteristically of *Bacteroides*, Bt contains a large complement of glycosylhydrolases enabling it to cleave a great number of the glycosidic bonds found in nature (Xu et al., 2004) which would undoubtedly give it a competitive advantage in its intestinal habitat.

*Bacteroides thetaiotaomicron* VPI-5482 produces OMVs that have been implicated to play a role in host metabolism (Rakoff-Nahoum et al., 2014), intracellular signaling (Stentz et al., 2014), and antimicrobial resistance (Stentz et al., 2015) as well as commensal microbial ecology and innate immunity (Hooper et al., 2003; Zakharzhevskaya et al., 2017). Bt-produced OMVs can promote colitis via the acquisition by and activation of host immune cells (Hickey et al., 2015), though the closely related *B. fragilis* can ameliorate host pathology by inducing enhanced regulatory T-cells and anti-inflammatory cytokine production (Shen et al., 2012). The internalization of *E. coli* OMVs by cultured polarized colonic epithelial cells shows that OMVs are internalized by host cells (José Fábrega et al., 2016), suggesting that metabolites present in OMVs could in principle be utilized for host metabolism. In addition to protein and polysaccharide delivery OMVs have been shown to mediate metabolic transfer in the form of inter-species carbon flux (Biller et al., 2014). While ^13^C-labeled fluxomics combined with metabolomics has been used to show metabolic activity within OMVs produced by enteropathogenic *Bacteroides fragilis* (Zakharzhevskaya et al., 2017), to date the role of OMVs in metabolic exchange between intestinal bacteria and their hosts has not been investigated.

Constraint-based genome-scale metabolic models (GEMs) have been used extensively in the analysis of bacterial and mammalian metabolism. The complete set of reactions known to be catalyzed in a particular organism can be formulated as a set of linear equations, a GEM, enabling the use of linear optimization techniques (including flux balance analysis, FBA) to tackle such problems as drug target identification (Raman et al., 2005), strain optimization for bioethanol production (Sengupta et al., 2013), and the elucidation of metabolic interactions during host infection by bacteria (Bordbar et al., 2010). FBA has been applied to multiple reconstructed metabolic networks of bacteria to show metabolic interactions between these bacteria when co-existing in a bacterial community in the human gut (Shoaie et al., 2013). Also an integrated model of the germ-free mouse and Bt metabolism has been created which captures the mutualistic nature of their metabolic interactions (Heinken et al., 2013). Heinken et al. (2013) showed the extent of the metabolic dependency between the two organisms, along with predictions for rescue of mouse growth phenotypes due to Bt metabolic activity. Recently metabolic reconstruction of over 750 gut-residing bacteria has been undertaken, paving the way for large-scale computational studies of metabolic interactions between the entire microbiota and the host (Magnúsdóttir et al., 2017). Additional applications of GEMs to the gut microbiota have recently been reviewed in detail (van der Ark et al., 2017). However, despite recent leaps in the ability to reconstruct and analyze such networks, even those of the most well-studied organisms are incomplete and limited by our biochemical knowledge. Unresolved impediments to the completion of such networks include uncharacterized, misannotated, or incompletely annotated enzymes, and when models are combined to study metabolic interactions the mechanisms of such interactions are not always clear.

In the present study, the small molecule content of OMVs produced by *B. thetaiotaomicron in vitro* has been investigated using a high coverage untargeted metabolomics analysis and *in silico* constraint-based modeling. A core set of metabolites has been identified that is packed into OMVs in two contrasting conditions. We have adapted and expanded an existing host–microbe constraint-based metabolic model to allow for the possibility of cross-kingdom metabolite transport via OMVs. By combining experimental data with FastGapFill (FGF) (Thiele et al., 2014), an algorithm for inferring missing reactions in incomplete metabolic networks, we have linked the observed metabolites in OMVs to both the Bt and mouse metabolic networks. By computational interrogation of this expanded model (using FBA) we have shown that the core set of available OMV metabolites is significantly enriched in *in silico* mouse-consumable metabolites. This metabolomic study and consequent *in silico* analysis offers compelling evidence that OMVs offer a potential conduit for metabolic interactions between gut bacteria and their mammalian hosts.

## Results and Discussion

### OMVs Contain a Core Set of Metabolites

**FIGURE 1:**
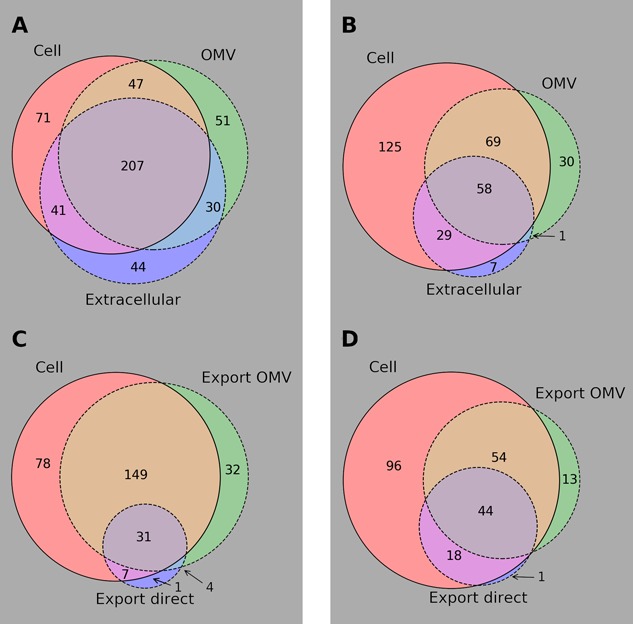
Summary of metabolomics results. Venn diagrams of metabolomic results for *B. thetaiotaomicron* and its OMVs grown *in vitro* in rich and defined media. Subfigures **(A)** and **(B)** show overlap of all identified metabolites in each of the compartments considered in RM and DM, respectively. Subfigures **(C)** and **(D)** show cell and OMV contents that could be mapped to KEGG metabolites along with those metabolites inferred to be exported directly to the medium between TP1 and TP2, in RM and DM, respectively.

Bt was cultured in rich medium (RM) and defined medium (DM), and samples were taken to assess its metabolite complement during exponential growth and shortly after entry into stationary phase (time points 1 and 2, respectively – TP1 and TP2) with the cellular, extracellular, and OMV contents under these different conditions analyzed by reverse phase (RP)/Ultrahigh Performance Liquid Chromatography-Tandem Mass Spectroscopy (UPLC-MS/MS) (ESI+) (-ESI) (Evans et al., 2014). OMV sizes were measured by nanoparticle analysis to be between approximately 40 and 700 nm in diameter. To assess the consistency of metabolite presence calls in each sample, the fraction of metabolites detected in all three replicates or no replicates in each sample was calculated. Over 93% of all measured metabolites in all samples were consistently present or absent in all replicates, indicating high consistency in measured replicates. In all 507 metabolites were identified by Metabolon in at least one of the conditions. Details of these metabolites can be found in Supplementary Table 1, which also contains details of what data are missing for each metabolite. **Figure 1** shows a summary of the observations of the metabolites during stationary phase in both RM (**Figure 1A**) and DM (**Figure 1B**); there is a lot of overlap in the contents of the three compartments in both media though there is an appreciable number of metabolites only seen in OMVs.

**Table 1** shows the number of metabolites in each super-pathway in each cellular and OMV sample analyzed. Strikingly similar numbers can be seen for the two time points in each medium, indeed of the 297 metabolites observed in DM at TP1 or TP2 over 90% are present at both time points (the figure is 84% in RM). This consistency is despite presumed depletion of the medium during growth and the switch to the stationary phase metabolic mode. The distribution of metabolites is similar between cell and OMV (in both media), apart from a large enrichment in lipids in OMVs with respect to cells. While 16% (15%) of the cellular metabolites are lipids in RM (DM), 28% (35%) of OMV metabolites are lipids. Although OMV lipids must somehow allow for the diversity of curvature required for the different sizes of OMVs observed *in vitro* and for very much higher surface-area-to-volume ratios than normal cells, there is no indication from this work why the repertoire of lipids should be so much greater than that of their originating cells. The difference between OMV content in rich (RM OMV) and defined (DM OMV) media was assessed. While many metabolites are seen solely in RM OMVs (181) a further 154 metabolites are observed in both, and only four metabolites seen solely in DM OMVs. This pattern of metabolite presence extends to the cell, only 10 of the 281 metabolites observed in cells grown in DM are absent in cells grown in RM. These results support the idea that rather than running differing modes of metabolism in both media, there is instead a core metabolism that runs in the cell producing consistent “core” metabolite pools and OMV contents. Given that the environment experienced by Bt *in vivo* is variable, but that the chyme entering the lower GIT is poor in nutrients (Spiller, 1994), the DM environment would appear to mimic more closely these *in vivo* conditions. The additional metabolites seen in the cell and RM OMVs are therefore supplementary to this core metabolism and could be by-products of the metabolic processes active in the cell during growth in this replete medium.

**Table 1:** A summary of the types of metabolites found by untargeted metabolomics in each cellular and OMV sample.

Super pathway	Defined medium			Rich medium		
	Cells		OMV (DM OMV)	Cells		OMV (RM OMV)
	6 h (TP1)	12 h (TP2)		2.5 h (TP1)	5 h (TP2)	
Amino acid	79	76	36	99	116	89
Carbohydrate	38	37	16	40	39	32
Cofactors and vitamins	29	29	5	30	30	15
Energy	8	8	6	8	9	8
Lipid	42	42	55	69	59	95
Nucleotide	53	51	20	64	65	42
Peptide	23	24	15	26	27	29
Xenobiotics	16	14	5	19	21	25
**Total**	**288**	**281**	**158**	**355**	**366**	**335**

*Each number represents the count of metabolites in each super pathway (based on KEGG super pathways). Cell samples are classified by time of sample (in hours) and all samples have their abbreviation in the text in brackets.*

An updated and extended metabolic model, denoted iMM_BT_OMV, was created to take into account the metabolomic data presented here. The number of compartments was increased by 3 to allow separate mouse and Bt cytosol/extracellular space, and the addition of OMVs as a potential metabolite transfer route. Two hundred and nineteen KEGG-mapped metabolites were added to the OMV compartment, requiring the addition of 438 “transport” reactions to represent the packing of the metabolites into OMVs by Bt and their potential uptake by mouse. Gap filling provided another 212 putative reactions in the Bt cell and 269 in the mouse cytosol amounting to 15% and 7% increases in the total number of reactions in each organism. Validation was undertaken by a manual search for catalyzing enzymes for those reactions in mouse that had substrates in the pool of available OMV metabolites.

Each metabolite in the Bt cell in the model was tested for essentiality for *in silico* growth in the DM using this model. This was done by preventing flux in all reactions involving each metabolite in turn, and running FBA to test whether growth was maintained when the metabolite was neither produced nor consumed. The *in silico* “essential” metabolites were therefore those which had to be either produced or consumed (or both) *in silico* to be consistent with experimental observations. This essentiality is not a prediction of presence in measurable amounts in the cell, as metabolic turnover in cells is very high and production and consumption can be finely balanced to eliminate the accumulation of unwanted (for instance toxic) small molecules.

Cell contents at TP2 are correlated with OMV contents in the RM (*p* = 0.015), but there is a much more pronounced relationship in DM (*p* = 5.9 × 10^-14^). There is not a correlation between the non-essential metabolites in RM cells and OMVs at TP2 (*p* = 0.054); however, in DM the correlation remains significant (*p* = 6.6 × 10^-11^). This appears counter-intuitive, as it might be expected that cells under nutrient stress would avoid accumulation of metabolites that were not necessary for survival of the cell. In this case the cells would drop pools of metabolites whose only (apparent) function is as contents of produced OMVs. In DM cell contents are correlated with *in silico* essential metabolites but there are still 78 observed small molecules which are not essential *in silico*, nor are they seen in DM OMVs. Forty-five of these are observed in RM OMVs, so a potential reason for their presence is in readiness for a change in nutrient availability at which point they can be released to OMVs.

Metabolites seen in OMVs in both media (“core” OMV metabolites) are significantly more likely to have been observed in Bt cells in RM (*p* = 4.6 × 10^-4^) than metabolites only observed in RM OMVs. Cells grown in RM maintain measurable pools of 112 metabolites that are also observed in OMVs produced by those cells. Sixty-four of these pools are depleted in cells grown in DM, but of those metabolites seven are maintained in OMVs produced in DM. Not only does this imply an important role for these seven metabolites, but also it indicates the control that Bt has over the small molecule content of released OMVs. For these metabolites at the very least, their presence in the OMV is not a by-product of their presence in the cell, but of specific packing into OMVs despite a lack of a measurable pool of metabolites from which to draw. Three of these metabolites have a role in osmoregulation and are therefore implicated in the stability of OMVs (Burg and Ferraris, 2008): arabitol, betaine, and genistein. Further, pilot NMR studies (unpublished data) have suggested that in RM betaine, mannitol and trehalose are among the most abundant OMV contents, though only betaine is seen in DM. The high stability of OMVs (Arigita et al., 2004; Kulp and Kuehn, 2010) could be at least partly explained by the presence of these molecules in their luminal space, along with other osmolytes observed such as carnitine, proline, and choline. Bt also limits the packing of some metabolites into OMVs, even when they are observed in the cells. There are 69 metabolites that are present in RM OMVs that are not exported to OMVs when cells are grown in DM, despite the cell maintaining measurable pools of those metabolites in the DM. The control exerted by Bt over its OMV small molecule contents, going as far as blocking available metabolites and packing metabolites with limited availability in the cell into OMVs must be for some biological purpose as it represents a significant investment of energy (Roier et al., 2016).

### Bt Preferentially Packs OMVs with Molecules Metabolized *in Silico* in the Mouse

To test the assertion that the core metabolites packed into OMVs are intended for the mammalian host, the predicted consumability of those metabolites by mouse (*in silico* according to iMM_BT_OMV) was calculated using FBA. For a metabolite to be useful to the host, the host must have an enzyme (or set of enzymes) to catalyze the conversion of that metabolite to a useful product. In order to enable unambiguous testing of *in silico* consumability of metabolites, only those metabolites which could be mapped to the KEGG database (Kanehisa et al., 2012), and therefore to KEGG reactions which were amenable to FBA analysis, were considered. To link the extracellular metabolomics data to internal Bt metabolism, metabolite export was calculated by observation of metabolite concentration changes between TP1 and TP2 in each extracellular sample. **Figures 1C,D** summarize the KEGG-mapped metabolites and their export from the bacterium in RM and DM, respectively.

For the *in silico* consumability analysis the integrated model was subjected to the availability of metabolites based on the Western Diet as defined by Heinken et al. (2013) and enrichment was calculated with respect to the proportion of consumable KEGG-mapped metabolites in the entire metabolomics dataset. In this environment there is a significant enrichment of consumable metabolites in the OMV (*p* = 0.008), whereas mouse-consumable metabolites are not significantly over-represented in the set of directly exported small molecules (*p* = 0.33). A lack of information on which metabolites are measurable in this analysis makes further statistical analysis difficult. It is, however, worth noting that while 62% of the metabolites producible by Bt (*in silico*) with nutrient availability as set above are consumable by mouse, 93.5% of the KEGG-mapped core OMV metabolites are consumable. These results are a clear indication that Bt preferentially packs its OMVs with metabolites that the mouse can metabolize.

To identify whether a particular pathway or set of pathways is targeted by Bt with its OMV contents, the distribution of core OMV metabolites in KEGG pathways was investigated by metabolite set enrichment analysis (Xia et al., 2015). The results show that OMV contents are not significantly enriched in any KEGG metabolic pathways (either in mouse or Bt) with respect to the metabolites imported to Bt, observed in Bt cells, or predicted essential *in silico*. It seems therefore that no particular pathways are targeted by Bt, so any specific metabolic response expected from the host or insufficiency in the host appears to be targeted only by individual metabolites.

Although the selections of medium for this study do not mimic conditions in the mammalian colon, they were selected for the purposes of establishing a baseline OMV metabolite content in defined minimal media. The RM was selected in order to establish the potential range of metabolites exportable via OMVs in replete conditions. *In vivo* the nutrient content of the colon is poor, but variable. Actual OMV contents *in vivo* are highly variable depending on available substrates and physiological conditions, but we hypothesize that they will contain a large fraction of the core metabolome, along with other condition-specific metabolites. We have also established a large set of metabolites that we now know can be packed into OMVs by Bt. Establishing exact *in vivo* OMV complements will require considerable further experimental work. We note, however, that our analysis shows a clear enrichment of mouse-consumable small molecules in the observed core metabolome indicating that our choices of medium are sufficient to begin probing the metabolic exchange between Bt and mouse via OMVs *in vivo*.

### OMV Metabolites Are Associated with Colonization of the Murine GIT

A previous study has identified a set of metabolites that discriminate between germ-free and conventionally reared mice (Claus et al., 2008), being absent in the former and present in the latter. In that study these metabolites were found in various tissues in the mouse including kidney, liver, and various sections of the GIT. The core OMV metabolites identified in this study is significantly enriched in these metabolites (*p* = 3*e*-68), containing 20 of the 25 metabolites that could be mapped between this study and that of Claus et al. (2008). A tantalizing interpretation of these observations is that Bt might directly supply these metabolites in appreciable quantities to the host via their OMVs. Further metabolomic experimentation on gnotobiotic mice colonized by Bt would be required to confirm this empirically. Metabolites that are directly exported by Bt are also significantly enriched in these discriminating metabolites (*p* = 5*e*-58), accounting for one additional metabolite of the 25.

All eight amino acids (classified as peptides) that were significantly different between GF- and conventionally reared mice in the study of Claus et al. (2008) are observed in OMVs in this study, and none are present in the DM itself, suggesting that Bt, one of the dominant members of the human intestine microbiota (Eckburg et al., 2005), could play a role in amino acid biosynthesis *in vivo*, using OMVs as the channel for their transport to the host.

### The Core OMV Metabolome Enables Putative Functional Annotation of Enzymes in Mouse

**FIGURE 2:**
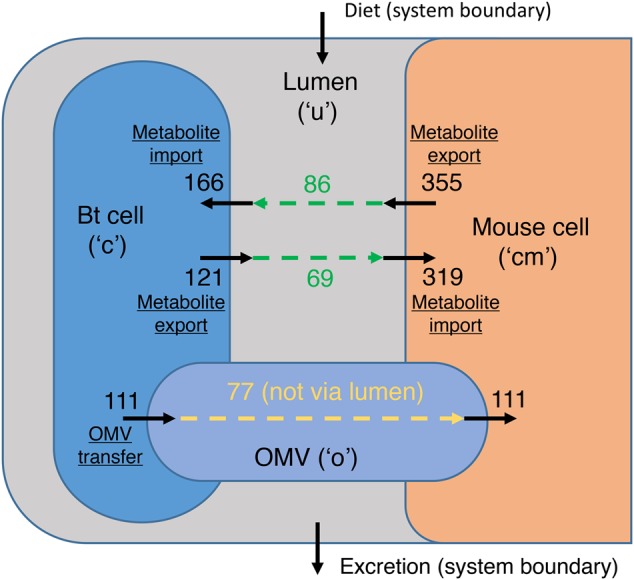
Summary of model expansion. The compartments of model iMM_BT_OMV that are affected by the presence of OMVs in the model. Numbers by solid arrows represent numbers metabolites that can cross compartment boundaries. Green dashed arrows represent the overlap between exported metabolites from one organism and imported metabolites to the other organism, along with the sizes of these overlaps. All OMV metabolites can potentially be transported from Bt to mouse. The cream dashed arrow represents the transfer from Bt to mouse via OMVs, and indicates the number of metabolites only transferrable via this route. The system boundary indicates how the model is connected to the dietary intake of nutrients and excretion from the GIT (via the lumen).

**Figure 2** shows the compartments that are affected by the addition of an OMV compartment to the expanded model (enabling metabolite transfer from Bt to mouse) and the numbers of metabolites that can be transferred between these compartments according to the metabolites observed in OMVs in either medium. It shows that while 69 metabolites that are directly exportable by Bt can be taken up by the mouse, OMVs enable the transport of a further 77, increasing the repertoire of potential metabolic interactions greatly. Gaps created by the addition of the OMV compartment in the expanded model iMM_BT_OMV were addressed using FGF (Thiele et al., 2014), which was run during the creation of the expanded model to find candidate reactions to link metabolites seen in the OMV with mouse metabolism.

In all 87 of the 104 consumable core OMV metabolites in the expanded model are *in silico* consumable in the original metabolic model iexGFMM_BΘ. FGF found a suitable set of reactions to add to the model to enable the connection of the remaining 17 to the mouse metabolic network. Two of these metabolites, creatinine and L-homoserine, exist in the original model mouse cytosol, but are not consumable in that model. The remainder do not take part in any known reactions in the mouse and are only convertible to a usable substrate by the addition of extra reactions. **Table 2** shows a list of the 17 metabolites for which reactions were inferred to be present in mouse by FGF.

**Table 2:** The proposed reactions by which metabolites present in the OMV could be connected to mouse metabolism.

BiGG ID	Common name	Reaction(s)	Producibility/ consumability	Required enzyme (EC number)	Enzyme in mouse (Gene ID)
13zdst	Erucic acid	Exporter (“13zdstt-_rev_e”)	P/C	–	–
1ag	1-Acylglycerol	R07293 (R)	P/C	Monoacylglycerol acylhydrolase	Lipe (16890.1)
3amp	Adenosine 3′-phosphate	R01562 (I)	NP/C	3′-AMP phosphohydrolase (3.1.3.6)	–
		R03537 (I)	P/NC	2′,3′-Cyclic AMP 3′-nucleotidohydrolase (3.1.4.16)	–
3c3hmp	Alpha-Isopropylmalate	R01213 (I)	P/NC	Acetyl-CoA:3-methyl-2-oxobutanoate C-acetyltransferase (2.3.3.13)	–
9z12zocdcya	Linoleate	R08177 (I)	NP/C	Linoleoyl-CoA hydrolase (3.1.2.2)	Acot2^∗^ (171210.1)
Achms	*O*-Acetyl -homoserine	R01287 (I)	NP/C	*O*-Acetyl -L-homoserine acetate-lyase (2.5.1.49)	–
		R01776 (I)	P/NC	Acetyl-CoA:L-homoserine *O*-acetyltransferase (2.3.1.31)	–
Crtn	Creatinine	R01884 (R)	P/C	Creatinine amidohydrolase (3.5.2.10)	–
g3pe	Glycerophospho-ethanolamine	R01470 (I)	NP/C	Glycerophosphohydrolase (3.1.4.2)	Gpcpd1 (74182.1)
Genistein	Genistein	R06553 (I^†^)	NP/C	Isoflavone synthase	–
hom__L	L-Homoserine	R01776 (I)	NP/C	Acetyl-CoA:L-homoserine *O*-acetyltransferase (2.3.1.31)	–
		R00175 (I^†^)	P/NC	*S*-Adenosyl -L-methionine hydrolase (3.3.1.2)	–
idt__L	L-Iditol	R07145 (R)	P/C	L-Iditol:NAD+ 2-oxidoreductase	Sord^∗^ (20322.1)
Malon	Malonate	R00743 (R)	P/C	Acetyl-CoA:malonate CoA-transferase (2.8.3.3)	–
Maltttr	Maltotetraose	R03801 (I)	NP/C	1,4-Beta-D-Glucan glucohydrolase (3.2.1.74)	–
		R03802 (I)	P/NC	1,4-Beta-D-Glucan glucohydrolase (3.2.1.74)	–
metsox_S__L	L-Methionine *S*-oxide	R02025 (I)	NP/C	L-Methionine:oxidized-thioredoxin *S*-oxidoreductase	Msrb2 and Msrb3 (76467.1 and 320183.1)
psphings	Phytosphingosine	R06525 (I)	P/NC	Sphinganine, ferrocytochrome b5:oxygen oxidoreductase	Degs2 (70059.1)
rbn__D	D-Ribonate	R01079 (R^†^)	P/C	D-Ribose:NADP+ 1-oxidoreductase (1.1.1.115)	–
saccrp__L	Saccharopine	R00715 (R)	P/C	N6-(L-1,3-dicarboxypropyl)-L-lysine:NAD+ oxidoreductase (1.5.1.7)	–

*Reactions are referenced by KEGG ID and proposed mouse genes include NCBI gene ID. Further abbreviations are as follows for properties of the reactions in mouse: R, reversible; I, irreversible; P, producible; NP, not producible; C, consumable; NC, not consumable. ^∗^Enzymes already present in the original model, but annotated with other roles; ^†^data from ModelSEED (Henry et al., 2010).*

**FIGURE 3:**
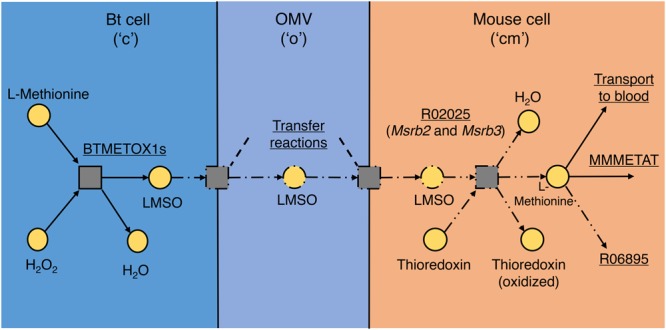
L-Methionine *S*-oxide (LMSO) transport and utilization pathway. The addition of reactions to model iMM_BT_OMV to represent transfer of LMSO by OMV from Bt to mouse and the enzyme found that could reduce LMSO to L-methionine in mouse. Squares represent reactions and circles represent metabolites, separated into their relevant compartments, “c,” “o,” and “cm,” in the model. Arrows show the direction of reaction flux; solid lines represent parts present in the original model, dash-single-dot lines represent parts added in OMV metabolism and dash-double-dot lines represent the reaction added during gap filling to link imported LMSO to mouse metabolism (by reduction). Reaction names and summaries are underlined.

**Figure 3** shows an example of reactions added due to the presence of a metabolite in the OMV, in this case L-methionine *S*-Oxide (LMSO). LMSO was observed in OMVs in both media so is predicted to be transferred to mouse, but there is no known existing reaction connecting LMSO to the rest of mouse metabolism. FGF predicted the reaction L-methionine:oxidized-thioredoxin *S*-oxidoreductase (KEGG ID: R02025) and consequently by database search two genes were found, the products of which together are predicted to constitute an enzyme catalyzing this reaction. L-Methionine is an essential amino acid and was shown by Heinken et al. (2013) to be competitively taken up by mouse and Bt *in silico*. However, the presence of *S*-oxidized L-methionine in OMVs produced in both media indicates that Bt does not compete directly with the host for L-methionine, produces it in measurable quantities inside the cell in both conditions and even exports it via OMVs, presumably with the intention of providing the host with this amino acid.

For all reactions added by FGF, reversibilities [initially calculated using eQuilibrator (Noor et al., 2012)] were checked against their curated KEGG reaction reversibilities. Several were inconsistent and in these cases the curated reversibility was adopted in the model. This resulted in two metabolites, alpha-isopropylmalate and phytosphingosine, becoming inconsumable in the mouse cytosol. In four cases FGF added two reactions for a DM OMV metabolite to make it consumable and in each case both reactions were irreversible, with one reaction producing the metabolite and one consuming it. Therefore, of the 17 DM OMVs metabolites that were made consumable in the FGF gap filling step, 15 were rendered consumable by the addition of just a single metabolic reaction per metabolite. For one of these, erucic acid, unblocking was achieved in the model through a transporter between the mouse cytosol and extracellular space (toward the lumen) rather than through integration into mouse metabolism. There is therefore no indication of how (and if) this metabolite connects with mouse metabolism, so it was not analyzed further.

The inference by gap filling of additional reactions to consume 14 extra metabolites in the updated model does not guarantee that these reactions can actually occur *in vivo* in the mouse so evidence for enzymes catalyzing these reactions was sought in MouseCyc (Evsikov et al., 2009) and the NCBI Gene database (NCBI Resource Coordinators, 2016). Five enzymes were found fitting five of the enzymatic roles required and are listed in the last column of **Table 2**. Lipe is annotated in the NCBI Gene database as a hormone-sensitive lipase, and we propose that it catalyses a monoacylglycerol acylhydrolase reaction. Acot2 is associated with reactions MMPTE2x, MMPTE3x, MMPTE4x, and MMPTE5x, a set of peroxisomal acyl CoA thioesterase reactions in the original mouse metabolic model, which share the EC number, 3.1.2.2, with the Linoleoyl-CoA hydrolase function proposed in this model. Gpcpd1 is annotated as a glycerophosphocholine phosphodiesterase in the Gene database, we propose that specifically it has a glycerophosphohydrolase function on glycerophosphoethanolamine.

Sord, associated in the model with the D-sorbitol dehydrogenase reaction MMSBTD_D2, is proposed additionally to have specificity for L-Iditol. The final proposed enzyme is heteromeric, consisting of Msrb2 and Msrb3 subunits which are annotated in the Gene database with a methionine sulfoxide reductase enzymatic function. We propose that this function is undertaken by this enzyme in the mouse (as illustrated in **Figure 3**). It should be noted that due to the potential for enzyme promiscuity these proposed functions are not intended to supersede previous annotations but as additional putative functions.

In addition, MouseCyc includes the *O*-acetyl-L-homoserine acetate-lyase reaction (KEGG:R01287) in the metabolic network of mouse, indicating that although it is not in the published model of mouse metabolism it is known with some confidence to be catalyzed in mouse, but that the relevant enzyme has not been identified to date. Although literature searches failed to find empirical evidence to support the functional inferences made here, it is striking that putative enzymes could be found for more than a third of the reactions predicted to be present.

### OMV Lipids Are Not Enriched with Lipids Found in Mouse Membranes

Twenty KEGG-identified lipids are contained in the lipid membrane of OMVs produced in DM and 12 of these are contained in the original mouse model (the mouse submodel of iexGFMM_BΘ), but this does not represent an enrichment of mouse lipids in OMVs with respect to all Bt lipids (the mouse shares 36 out of the 78 lipids produced by Bt according to iexGFMM_BΘ). Fourteen of the lipids observed at TP2 in Bt cells grown in DM are contained in the OMV membrane, but of the additional six produced by Bt specifically for OMVs (i.e. not seen in cell samples) only one is present in the original mouse model. OMV lipid content is therefore not driven by a requirement for similarity to the host lipid membrane. Without further experimental investigation, it is not possible to say which subset of the observed lipid complement in Bt cells is part of the outer membrane of Bt, but this addition of six further lipids to OMVs where they are not present in the cell indicates that Bt controls the lipid complement of OMVs for some purpose.

These extra lipids are not significantly shorter than those in Bt cells, nor are they significantly more saturated than the lipids present in those cells, so they do not appear to be added to accommodate the extra curvature of OMVs due to their physical or chemical properties. Other possible mechanisms driving membrane curvature and OMV biogenesis have been reviewed previously (Schwechheimer and Kuehn, 2015). There is evidence that internalization of *E. coli* OMV contents in Caco-2 cells can be achieved via membrane fusion (José Fábrega et al., 2016), but the fate of the non-mouse lipid content of the OMVs on fusion with host cells remains unknown. Every lipid found in OMVs from DM is also present in OMVs from RM, reflecting the additive nature of OMV packing by Bt when provided with additional nutrients. Other possible reasons for the observed lipids in OMVs could be as artifacts of sample preparation and storage, or as degradation products of active enzymes within the vesicles. Investigation of this second possibility will require proteomic analysis to identify lipid-degrading enzymes in these vesicles.

One hundred and twenty of the 339 metabolites observed in OMVs could not be mapped to KEGG IDs including 46 core OMV metabolites, so their metabolic impact could not be analyzed through FBA; however, there is information from Metabolon about their classification in (KEGG-based) super pathways. These 46 include 26 lipids and 14 dipeptides. Including these data with the KEGG-mapped core metabolite data, lipids make up 21 of the 29 core metabolites that are not found in Bt cells during growth on DM. The absence from the KEGG database of such a high proportion of lipids indicates that lipids are under-represented in this reaction database. Conversely, all 15 of the dipeptides in the core metabolite set are seen in Bt cells as well, but it is currently unclear what significance this has for the function of the dipeptides.

### Challenges in Elucidating Host–Microbe Metabolic Interactions via OMVs

Most digestion initiates in the small intestine before the chyme is transferred to the colon, the segment of the GIT in which Bt mostly resides. Dietary differences between individuals and the pre-distal digestion would both have a large impact on nutrient availability in the colon and consequently an impact on the details of the consumability and producibility of metabolites by Bt. Although DM has been used to grow Bt for this analysis, it is not fully representative of the conditions and nutrient supply in the GIT. More precise specification of the availability of nutrients to Bt *in vivo* requires further experimentation, which would help to characterize better the OMV-mediated metabolic transfers from Bt to mouse.

In this study we have presented good evidence that Bt packs OMVs with metabolites that are targeted at the mammalian host, but several large questions remain unanswered from this analysis. Due to the lack of absolute quantification of OMV contents it is difficult to estimate the size of the impact that these metabolites might have on host cell metabolism. Many contents of the OMVs have been observed by Claus et al. (2008) in a mouse host only when bacteria are present in its GIT, but this is not sufficient to deduce their source to be OMVs produced by these bacteria. On the other hand, OMVs are known to be continuously produced by Gram-negative bacteria. OMV numbers were measured by nanoparticle tracking analysis, and found to be at similar levels [3 × 10^11^ (RM) and 5 × 10^11^ (DM) particles when adjusted to the total number]. Therefore, lack of nutrient availability does not appear to restrict OMV production, and the potential of OMVs to be metabolic mediators.

The inability to quantify the metabolites also means that it is impossible to determine which, if any, of the metabolites dominate the OMV, and which are only present in trace amounts. It therefore remains an open question to what extent and in what way mammalian metabolism is impacted by the small molecule content of OMVs. Discussion of specific metabolites here is therefore limited to their general classification and consumability in the mouse, and further investigation will be required to establish the exact role that OMVs take in mediating metabolism between host and bacterium. Analysis of OMVs from other prominent bacterial species in the human GIT would offer further insights into this conventionally overlooked route for metabolite exchange in the human gut.

### Applications to Modeling Human–Microbe Co-metabolism

Several attempts have been made to date to capture co-metabolism of host and microbe in the GIT in a systematic and representative way, in a model that is amenable to computational analysis (Heinken et al., 2013; Shoaie et al., 2013; Shoaie and Nielsen, 2014; Magnúsdóttir et al., 2017). An extension of these models to a human–microbe system has been undertaken wherein the idea of co-metabolism, viewing the GIT as just another organ in a model of human metabolism, is formalized in a constraint-based metabolic model (Heinken and Thiele, 2015). We have found that adding OMVs to such models would allow for a more comprehensive representation of the link between the GIT microbial “organ” and the rest of the human body.

### Conclusion

An investigation of the metabolomic content of Bt and the OMVs it produces, combined with FBA of an integrated mouse-Bt metabolic model, has shown that Bt controls the metabolite complement of OMVs and that the resulting metabolite profile is enriched for metabolites that its murine host can metabolize. Furthermore, these OMVs are greatly enriched in metabolites implicated in the distinctive metabolic profiles of conventionally reared mice as opposed to germ-free mice, whereas many of these metabolites are not excreted directly by Bt. These data illustrate the potentially crucial role OMVs play in metabolic interactions between bacterium and host over and above their established roles in immune modulation and in the breakdown of host-indigestible glycans. While this represents a step toward a greater understanding of these interactions, a lack of *in vivo* data means that conclusions about the specific effects of OMVs on host metabolism cannot yet be made. We do, however, present several mouse enzyme function predictions which if borne out would show that exogenous metabolite uptake from Bt (and presumably many other bacterial species present in the GIT) plays a significant role in host (mouse) metabolic evolution. The expanded metabolic model presented here should enable more realistic simulations of the mouse-Bt metabolic system taking these additional points of metabolic contact into account. The lipid content of OMVs appears not to be enriched for lipids present either in the murine large intestine or in mouse tissues in general (as inferred *in silico*), leaving the difference between the Bt lipid content and that of its OMVs unexplained. Also, as with many metabolomics experiments to date, these data throw a spotlight on the incompleteness of both metabolite and reaction databases and also of even recently curated metabolic models; the number of lipids unidentified in these databases shows that this is particularly the case in lipid metabolism. Together these analyses offer new and potentially important insights into a crucial aspect of host–microbe interactions that had thus far remained unexamined.

## Materials and Methods

### Sample Preparation

#### Bacterial Cell and Medium Extracts

The bacterium *Bacteroides thetaiotaomicron* VPI-5482 was grown anaerobically at 37°C with agitation using a magnetic stirrer in either Brain Heart Infusion medium (Oxoid/Thermo Fisher, Basingstoke, United Kingdom) with 0.001% hemin (rich medium, “RM” or in *Bacteroides* DM (“DM,” see **Table 3**) adapted from Martens et al. (2008).

**Table 3:** The concentrations of all small molecules present in the *Bacteroides* Defined Medium (DM) used to grow *Bacteroides thetaiotaomicron*.

Compound	Concentration (pH 7.4)
KH_2_PO_4_	100 mM
NaCl	15 mM
(NH_4_)_2_SO_4_	8.5 mM
MgCl_2_	0.1 mM
L-Cysteine	4.1 mM
Vitamin B12	3.7 × 10^-7^ mM
Hematin	0.0015 mM
Glucose	0.5% w/v
CaCl_2_	50 μM
Histidine	0.2 mM

For each medium 3 culture flasks were grown independently for each time point. Cells from overnight cultures were rinsed once with phosphate-buffered saline (PBS) and used to inoculate 200 ml fresh medium in 500 ml flasks at an OD of 0.05. After 2.5 h in RM and 6 h in DM (OD approximately 0.5, mid-exponential phase), 5 h in RM and 12 h in DM (OD approximately 3.0, early stationary phase), the cell culture (60 ml for mid-exponential phase and 10 ml for early stationary phase) was collected using 5 ml syringes fitted with a 13 mm diameter syringe filter with a 0.22-μm pore size hydrophilic PTFE membrane (IC Millex^®^ – LG, Millipore) and filled with pre-cooled 10–15 stainless steel spheres (5 mm diameter) at -20°C for rapidly cooling the broth for subsequent analysis of extracellular material (Mashego et al., 2006). Two hundred microliters of samples was collected in all cases. Sterile handling of the samples was confirmed by plating of one of the six samples in BHI–hemin agar. The cell culture was immersed directly into liquid N_2_, followed by thawing in ice to keep the cell suspension at 0°C. The suspension was later centrifuged to separate biomass from the supernatant at 5000 × *g*, 15 min, 4°C, and rinsed once in the same volume of ice-cold PBS. After removing the supernatant, the cell pellets were snap frozen in liquid nitrogen and stored at -80°C before extraction. The dry weight measurement was performed on cell pellets obtained from 30 ml cultures previously washed with one volume of PBS. Although OMVs would have remained in the supernatant, their concentration is small enough that their contribution in these conditions to metabolomics signal is negligible.

#### Bacterial OMV Extracts

In order to obtain a sufficient quantity of OMVs to detect a metabolomic signal, 2 l of RM (three culture flasks grown independently) and DM (three culture flasks grown independently) were inoculated with overnight culture of *B. thetaiotaomicron* at an initial OD of 0.0005 and 0.05, respectively. The OMV extraction was performed as previously described (Stentz et al., 2014) with slight modifications. After 16 h (OD approximately 4.0), the cell cultures were rapidly cooled in a manually shaken ice bath. The cultures were then centrifuged at 5000 × g for 15 min at 4°C, and the supernatants filtered through a 0.22-μm Steritop filtration unit (Millipore, Billerica, MA, United States) to remove debris and cells. The sterility of the filtrate containing the vesicles was confirmed by plating onto BHI–hemin agar. OMVs in the 2 l filtrates were concentrated by molecular weight (100 kDa MWCO, Vivaflow 200, Sartorius) down to 2 ml, diluted by addition of 1 l of ice-cold PBS, pH 7.4, and the suspensions were filtered and concentrated again, down to 9 ml. The 9 ml retentate was ultracentrifuged [150,000 × *g* for 2 h at 4°C in a Ti70 rotor (Beckman Instruments)]. The supernatant was completely removed using a vacuum pump and a needle and the OMV pellets were snap frozen in liquid nitrogen and stored at -80°C before extraction.

### Nanoparticle Analysis

Videos were generated using a Nanosight nanoparticle instrument to count OMV numbers in each OMV sample. The population of OMVs (moving due to Brownian motion) in the sample was recorded and their motion was measured. Within a specially designed and constructed laser illumination device mounted under a microscope objective, OMVs in the sample passing through a laser beam path were recorded as small rapidly moving points of light (for sub-wavelength sized particles these are simply scattering centers). The motions of these OMVs were tracked to determine their velocity and numbers. Simultaneous measurement of the mean squared displacement of each OMV tracked, the particle diffusion coefficient (*D_t_*), and hence sphere equivalent hydrodynamic radius (*r*_h_) were determined using the Stokes–Einstein equation,

(1) $$\begin{array}{c} D_{t}\;=\;\frac{k_{B}T}{6\pi \eta r_{h}}, \end{array}$$

where *k*_B_ is the Boltzmann’s constant, *T* is the temperature, and *η* is the solvent viscosity.

### Metabolomics

Samples were subject to metabolite extractions and analysis by RP/UPLC-MS/MS (ESI+) (-ESI), and subsequent bioinformatics data processing (Metabolon Inc., Durham, NC, United States). Details of the methods used have been published (Evans et al., 2014), and an outline of the process is provided here.

#### Sample Preparation

Samples were prepared using the automated MicroLab STAR^®^ system from Hamilton Company. DL-2-fluorophenylglycine, tridecanoic acid, d6-cholesterol, and 4-chlorophenylalanine were utilized as recovery standards. To remove protein, dissociate small molecules bound to protein or trapped in the precipitated protein matrix, and to recover chemically diverse metabolites, proteins were precipitated with methanol under vigorous shaking for 2 min (Glen Mills GenoGrinder 2000) followed by centrifugation. The resulting extract was divided into five fractions: two for analysis by two separate RP/UPLC-MS/MS methods with positive ion mode electrospray ionization (ESI), one for analysis by RP/UPLC-MS/MS with negative ion mode ESI, one for analysis by HILIC/UPLC-MS/MS with negative ion mode ESI, and one sample was reserved for backup. Samples were placed briefly on a TurboVap^®^ (Zymark) to remove the organic solvent. The sample extracts were stored overnight under nitrogen before preparation for analysis.

#### Ultrahigh Performance Liquid Chromatography-Tandem Mass Spectroscopy (UPLC-MS/MS)

All methods utilized a Waters ACQUITY ultra-performance liquid chromatography (UPLC) and a Thermo Scientific Q-Exactive high resolution/accurate mass spectrometer interfaced with a heated electrospray ionization (HESI-II) source and Orbitrap mass analyzer operated at 35,000 mass resolution. The sample extract was dried then reconstituted in solvents compatible to each of the four methods. Each reconstitution solvent contained a series of standards at fixed concentrations to ensure injection and chromatographic consistency. One aliquot was analyzed using acidic positive ion conditions, chromatographically optimized for more hydrophilic compounds. In this method, the extract was gradient eluted from a C18 column (Waters UPLC BEH C18-2.1 × 100 mm, 1.7 μm) using water and methanol, containing 0.05% perfluoropentanoic acid (PFPA) and 0.1% formic acid (FA). Another aliquot was also analyzed using acidic positive ion conditions; however, it was chromatographically optimized for more hydrophobic compounds. In this method, the extract was gradient eluted from the C18 column using methanol, acetonitrile, water, 0.05% PFPA, and 0.01% FA and was operated at an overall higher organic content. Another aliquot was analyzed using basic negative ion optimized conditions using a separate dedicated C18 column. The basic extracts were gradient eluted from the column using methanol and water, however, with 6.5 mM Ammonium Bicarbonate at pH 8. The fourth aliquot was analyzed via negative ionization following elution from a HILIC column (Waters UPLC BEH Amide 2.1 × 150 mm, 1.7 μm) using a gradient consisting of water and acetonitrile with 10 mM ammonium formate, pH 10.8. The MS analysis alternated between MS and data-dependent MS^n^ scans using dynamic exclusion. The scan range varied slighted between methods but covered 70–1000 m/z. Raw data files are archived and extracted as described below.

### Bioinformatics

The informatics system consisted of four major components, the Laboratory Information Management System (LIMS), the data extraction and peak-identification software, data processing tools for QC and compound identification, and a collection of information interpretation and visualization tools.

Raw data were extracted, peak-identified, and QC processed using Metabolon’s in-house web-service platform. Compounds were identified by comparison to library entries of purified standards or recurrent unknown entities. Metabolon maintains a library based on authenticated standards that contains the retention time/index (RI), mass to charge ratio (*m/z*), and chromatographic data (including MS/MS spectral data) on all molecules present in the library. Biochemical identifications were based on three criteria: retention index within a narrow RI window of the proposed identification, accurate mass match to the library ±10 ppm, and the MS/MS forward and reverse scores between the experimental data and authentic standards. The MS/MS scores were based on a comparison of the ions present in the experimental spectrum to the ions present in the library spectrum. While there may be similarities between these molecules based on one of these factors, the use of all three data points was used to enable differentiation of such biochemicals. More than 3300 commercially available purified standard compounds have been acquired and registered into LIMS for analysis on all platforms for determination of their analytical characteristics.

A variety of curation procedures were carried out to ensure that a high-quality data set was made available for statistical analysis and data interpretation. The QC and curation processes were designed to ensure accurate and consistent identification of true chemical entities, and to remove those representing system artifacts, mis-assignments, and background noise. Proprietary Metabolon visualization and interpretation software were used to confirm the consistency of peak identification among the various samples. Library matches for each compound were checked for each sample and corrected if necessary. Data normalization was performed according to the protein concentrations measured with the Bradford assay (cells, extracellular milieu, and vesicles).

Due to the nature of the above methods, metabolite concentrations in similar conditions (e.g., different time points in the same medium) are comparable. This enabled relative concentrations to be calculated between such samples.

### Data Analysis

#### Metabolomics Analysis and Interpretation

Metabolites were inferred to be present for a particular time point (TP1 or TP2), OMV sample, or medium (DM or RM) if they were measured in at least two of the three relevant replicates. Metabolite presence in each sample is detailed in Supplementary Table 1. FBA as detailed below was performed using only those metabolites that could be mapped to a KEGG ID, to ensure compatibility with pathway modeling. Where a KEGG ID had not been assigned by Metabolon to an entry in the list of metabolites, a KEGG ID was sought manually by reference to ChEBI (Hastings et al., 2013) and Pubchem (Kim et al., 2016), with any metabolites not identifiable by KEGG ID excluded from the analysis. Significant changes in metabolite concentrations in the media with respect to initial metabolite concentrations (from pre-inoculation blanks) were inferred by a one-sample two-tailed *T*-test. Multiple testing was accounted for using the Benjamini–Hochberg procedure to control false discovery rate with a significance threshold of 0.05 (*q* = 0.05) was used.

#### Update and Expansion of iexGFMM_BΘ

To map all the potential biosynthetic pathways involved in production of OMV metabolites to the combined Bt/mouse metabolic model, the complete list of KEGG-mapped metabolites found in either OMV sample was used in creating the updated and extended model of the system. The KEGG database of reactions (Kanehisa and Goto, 2000) was used as the set of candidate reactions for the expansion of both the mouse and Bt metabolic networks to fill gaps in the metabolic model. All inferences concerning each metabolite considered in this work can be found in Supplementary Table 1. All *in silico* GEM reconstruction, modeling, and analysis was done in Python using the COBRApy constraint-based modeling package (Ebrahim et al., 2013), except for the application of FGF (Thiele et al., 2014) which is implemented in the COBRA Toolbox v3.0 for MATLAB (Heirendt et al., 2017).

The expanded integrated Bt/mouse GEM was based on model iexGFMM_BΘ (Heinken et al., 2013). Before expansion of the model, several changes were made to allow the application of FGF (see below) and to bring it up to date with current nomenclature. Reactions and metabolites associated with the mouse cytosol were moved from the generic cytosol compartment “c” into a mouse-specific cytosol compartment “cm.” Similarly reactions and metabolites associated with the close neighborhood of mouse cells (the “extracellular” compartment) were moved from “e” to “em.” All metabolite IDs were updated in line with their current corresponding entries in the BiGG Models database (King et al., 2016).

A new compartment named “OMV” with ID “o” was added to the integrated model. When analysis was done in a specific medium, the reactions corresponding to metabolites that were not present in OMVs in that medium had their flux bounds set to 0. To map the metabolites (with KEGG IDs) added to the model, a set of correspondences between KEGG and BiGG IDs was required. This was adapted from the dictionary that accompanies the FGF software, with BiGG IDs updated and duplicate entries removed. Further ID mappings were sought manually for all KEGG IDs not included in the original dictionary using MetaNetX (Ganter et al., 2013) and ChEBI. Although the Bt model contains a “cytosol” compartment, it does not in fact differentiate between cytosolic and periplasmic reactions, so the Bt “cytosol” compartment is referred to as the Bt “cell” to make this explicit. If the OMV metabolites were not found in either the Bt cell (“c”) or the mouse cytosol (“cm”) they were added, as well as to the OMV compartment (“o”) (**Figure 2**). For each of these metabolites a pair of reactions were added, a transport reaction from “c” to “o” and a transport reaction from “o” to “cm.” These reactions were all set as irreversible to represent the flow of material: packed into OMVs by Bt, then released and taken up by mouse epithelial cells.

The inclusion of the OMV and other inferred reactions added a large number of gaps to the model, whereby metabolites were not producible or consumable in the mouse cell and/or in the Bt cell. FGF (Thiele et al., 2014) was used to connect up these metabolites to the rest of the metabolic network in such a way as to add the fewest reactions possible (from a complete list of KEGG reactions downloaded from the KEGG database). In short, FGF constructs a global model by adding a universal set of reactions (in this case all KEGG reactions) to the input metabolic model, then runs the FASTCORE algorithm (Vlassis et al., 2014) which iteratively computes by linear optimization a flux-consistent model comprising of all model reactions and a minimal set of added “universal reactions.” This is a fast algorithm, but is limited by the completeness and accuracy of the input metabolic network and database.

Since reaction downloads from KEGG do not contain reversibility information, eQuilibrator (Noor et al., 2012) was used to computationally estimate the reversibility of every KEGG reaction. FGF was applied individually to the mouse cytosol and the Bt cell to fill the gaps in both the biosynthetic pathways of the OMV metabolites in the bacterium and their consequent uptake and metabolism in the host cell.

For validation of all reactions that enabled the consumption (and production) of these metabolites reversibility and thermodynamically favorable direction in the model were manually checked against the KEGG database. Where this information contradicted the reversibility inference from eQuilibrator the curated KEGG reversibility was used. To find candidate enzymes for the reactions consuming these metabolites in the mouse, the EC number of each one was submitted to MouseCyc (Evsikov et al., 2009) to determine whether an enzyme had previously been identified for this reaction in mouse and if no enzyme was found, the NCBI Gene database (NCBI Resource Coordinators, 2016) was searched using the string “mus musculus [organism] x.x.x.x” where “x.x.x.x” is the relevant EC number in order to identify genes with a relevant annotation. Both updated reversibility and inferred enzyme functions were then added to the expanded model. If a gene found in this way was already in the model (annotated as an enzyme component catalyzing another reaction) an annotation was appended to the existing annotation. The expanded model is available in SBML format in the Supplementary Material.

#### *In Silico* Metabolite Analysis

The ability of Bt and mouse to both produce and consume each metabolite present in OMVs was determined by FBA. To enable the analysis of each organism independently, the other organism was blocked by setting the upper and lower bounds of all relevant exchange reactions between the lumen and that other organism to zero. Thus any metabolite would have to be produced or consumed by the host or bacterial organism, rather than be transported to or from the other organism. All OMV reactions were also limited to 0 flux during the producibility/consumability analysis to ensure assessment of endogenous production and consumption. A source or sink for each metabolite was added to the model enabling the distinction between being able to produce and being able to consume the metabolite; this source or sink reaction was set as the objective and maximized. Adding a source reaction was used to test whether the metabolite could be consumed and adding a sink reaction was used to test whether it could be produced. Input fluxes of metabolites representing food consumption were set according to the *in silico* Western Diet detailed by Heinken et al. (2013).

Assessment of the requirement for metabolite presence (even if not in detectable amounts) in the *in silico* Bt cell in iMM_BT_OMV was done using FBA. The Bt section of the integrated model was used to model *in vitro* growth. Exchange reactions for metabolites into the extracellular space around Bt were set to a lower flux bound of -100 g/gDW/h for all components of each medium. For each metabolite inferred from the metabolomics data to be imported by Bt *in vitro* a source reaction was created for that metabolite with a small non-zero minimum flux, to force the model solver only to return solutions that fit the qualitative metabolite uptake profile seen *in vitro*. Since the RM is not fully defined, but includes large numbers of metabolites, the lower bounds of all exchange reactions were set to -100 g/gDW/h for this medium. The set of reactions containing each metabolite in the Bt cell was then removed in turn, and the model optimized for biomass growth. If the growth achieved by optimization was below a cutoff of 0.1 h^-1^, then that metabolite was inferred to be produced by necessity by Bt in the course of growth.

#### Statistical Testing

Where enrichment analysis or comparisons were done for any differences in attributes (presence, flow, consumability, etc.) between two sets of metabolites, a χ^2^ test was used to calculate a *p*-value for enrichment (the probability that under the null hypothesis that the χ^2^ value would be greater than or equal to the empirical χ^2^ value), and the significance cutoff value used for all of these analyses was *p* = 0.05.

## Author Contributions

Conceptualization: SC, TW, MS, RS, and WB. Methodology: TW, RS, GLG, and WB. Software: WB. Formal analysis: WB. Investigation: RS. Writing – original draft: WB and RS. Writing – review and editing: WB, RS, GLG, MS, SC, and TW. Project administration: TW. Funding acquisition: MS and SC.

## Conflict of Interest Statement

MS is a Director and Shareholder in Equinox Pharma Ltd., which employs chemoinformatics and bioinformatics for drug discovery and services. The other authors declare that the research was conducted in the absence of any commercial or financial relationships that could be construed as a potential conflict of interest.
